# Prognostic significance of soluble PD-L1 in prostate cancer

**DOI:** 10.3389/fimmu.2024.1401097

**Published:** 2024-07-11

**Authors:** Margarita Zvirble, Zilvinas Survila, Paulius Bosas, Neringa Dobrovolskiene, Agata Mlynska, Gintaras Zaleskis, Jurgita Jursenaite, Dainius Characiejus, Vita Pasukoniene

**Affiliations:** ^1^ Laboratory of Immunology, National Cancer Institute, Vilnius, Lithuania; ^2^ Institute of Biosciences, Life Sciences Center, Vilnius University, Vilnius, Lithuania; ^3^ Department of Oncourology, National Cancer Institute, Vilnius, Lithuania; ^4^ Department of Immunology and Bioelectrochemistry, State Research Institute Centre for Innovative Medicine, Vilnius, Lithuania; ^5^ Department of Chemistry and Bioengineering, Vilnius Gediminas Technical University, Vilnius, Lithuania; ^6^ Department of Pathology and Forensic Medicine, Institute of Biomedical Sciences, Faculty of Medicine, Vilnius University, Vilnius, Lithuania

**Keywords:** prostate cancer, soluble PD-L1 and PD-1, biomarkers, prognosis prediction, immune cells

## Abstract

**Purpose:**

The aim of this study was to assess the role of sPD-L1 and sPD-1 as potential biomarkers in prostate cancer (PCa). The association of the values of these soluble proteins were correlated to the clinical data: stage of disease, Gleason score, biochemical recurrence etc. For a comprehensive study, the relationship between sPD-L1 and sPD-1 and circulating immune cells was further investigated.

**Methods:**

A total of 88 patients with pT2 and pT3 PCa diagnosis and 41 heathy men were enrolled. Soluble sPD-L1 and sPD-1 levels were measured in plasma by ELISA method. Immunophenotyping was performed by flow cytometry analysis.

**Results:**

Our study’s findings demonstrate that PCa patients had higher levels of circulating sPD-L1 and sPD-1 comparing to healthy controls (p < 0.001). We found a statistically significant (p < 0.05) relationship between improved progression free survival and lower initial sPD-L1 values. Furthermore, patients with a lower sPD-1/sPD-L1 ratio were associated with a higher probability of disease progression (p < 0.05). Additionally, a significant (p < 0.05) association was discovered between higher Gleason scores and elevated preoperative sPD-L1 levels and between sPD-1 and advanced stage of disease (p < 0.05). A strong correlation (p < 0.05), between immunosuppressive CD4+CD25+FoxP3+ regulatory T cells and baseline sPD-L1 was observed in patients with unfavorable postoperative course of the disease, supporting the idea that these elements influence each other in cancer progression. In addition to the postoperative drop in circulating PD-L1, the inverse relationship (p < 0.05), between the percentage of M-MDSC and sPD-L1 in patients with BCR suggests that M-MDSC is not a source of sPD-L1 in PCa patients.

**Conclusion:**

Our findings suggest the potential of sPD-L1 as a promising prognostic marker in prostate cancer.

## 1 Introduction

Prostate cancer (PCa) is still the second most prevalent type of cancer among men worldwide (1). Prostate specific antigen (PSA) is a widely used marker of diagnosis and prognosis in PCa, however there is evidence that changes in its levels are not related to survival outcomes (2) and PSA is often used mainly because of the lack of useful predictive markers (3).

Recently, soluble checkpoints PD-L1 and PD-1 (sPD-L1 and sPD-1), whose precursors are membrane bound PD-L1 and PD-1, have been the subject of intense research for their prognostic and predictive value in various cancers (4, 5). The dynamic alterations of membranous PD-L1 in the circulatory system, including sPD-L1 and other forms of PD-L1, are attributed to liquid biopsy technique (6).

Numerous cancer types have been found to have elevated sPD-L1 protein levels (7). A growing body of evidence revealed that patients with solid tumors and higher levels of soluble PD-L1 in their peripheral blood, have a significantly worse outcomes; this suggests that high levels of sPD-L1 could be a biomarker for poor prognosis (8, 9). In the meantime, patients with a variety of malignancies have higher levels of sPD-1 in their blood and pretherapeutic increase is associated to higher risk of cancer developing, the progression of the disease and a worse result, on the other hand, a stable or elevated sPD-1 levels following cancer treatment have been linked to better outcomes (5, 10, 11).

The origin of sPD-L1 remains unknown, as it could potentially originate from different sources, such as tumor cells (12, 13) and surrounding immune cells (7, 14) in particular, myeloid derived suppressive cells (MDSC) may serve as a natural source of sPD-L1 (15). Meanwhile human macrophages have been shown to express sPD-1 (16), other potential source of circulating sPD-1 is natural killer (NK) cells (17).

PCa is generally considered as an immunologically cold tumor with low PD-L1 expression, poor infiltration of immune T cells and with predominant immunosuppressive tumor microenvironment (TME) (18–23). T-regulatory cells (Tregs) and MDSC are prevailing immunosuppressive cells found within the TME (24) as well as in peripheral blood (25) of PCa. Insights into the interaction between cancer and the immune system may provide additional aspects of tumor development.

Despite the success of immunotherapy in other solid tumors, PCa treatment has shown limited response, particularly to single-agent checkpoint inhibition (20, 26). Radical prostatectomy (RP) and radiotherapy are the two most effective treatments for PCa that is primarily localized (27). The potential of sPD-L1 and sPD-1 as biomarkers for predicting treatment efficacy is suggested by changes in their levels following specific treatments, such as surgery, radiotherapy, and immunotherapy (5, 11). Even though PCa does not exhibit prominent PD-L1 expression, some scientific studies declares that sPD-L1, rather than membranous PD-L1, effectively predicts prognosis in other tumors (28).

Considering PCa as an immunologically cold tumor, the role of soluble PD-L1 and PD-1, and especially their association with peripheral blood immune cells, has not yet been thoroughly investigated in PCa tumors. The aim of this study was to evaluate the relationship between soluble PD-L1 and PD-1 molecules in the plasma of prostate cancer patients and their correlation with the clinical course of the disease. Additionally, we aimed to explore the association between soluble PD-L1 and PD-1 receptors and the immune status of patients. By unraveling these connections, our goal is to identify potential biomarkers that can inform the clinical progression of prostate cancer and shed light on the immune responses, paving the way for more targeted and personalized therapeutic interventions.

## 2 Materials and methods

### 2.1 Patients and healthy subjects

The study was approved by the Regional Review Board (Vilnius, Lithuania, 158200–17-928–442). All research methods were carried out in accordance with the relevant Lithuanian national guidelines and regulations. Written information about the study was provided to each study participant and written consent was obtained to participate in the study.

The inclusion criteria for patients were as follows (1) pT2 and pT3 PCa diagnosis. Exclusion criteria were as follows (1) history of other malignancies diagnosis or treatment; (2) androgen deprivation therapy (ADT), radiation therapy or chemotherapy prior to surgery and three months after surgery; (3) inflammatory conditions, immunosuppressive interventions, or autoimmune disease presence; (4) perioperative blood transfusions; (5) preoperative or postoperative white blood cell count exceeding 10,000 µL−1 (up to three months post-surgery); (6) abnormal levels of liver enzymes, glomerular filtration rate, C-reactive protein, or bilirubin, as previously described in preceding study involving the same cohort of patients (29).

All patients were followed up through clinic visits for 30 months following radical prostate excision. The starting point for follow-up was postoperative day 1. Endpoint events were as follows (1) biochemical recurrence (BCR); (2) failure of postoperative PSA to decrease to target < 0.2 ng/ml value; (3) the need of additional radiotherapy. Progression free survival (PFS) was defined as all above mentioned events.

Circulating preoperative and postoperative sPD-L1 and sPD-1 levels in PCa patients were compared with clinical-pathological data: Gleason score, biochemical recurrence (BCR), changes of prostate cancer antigen (PSA), need of radiation therapy, prostate cancer stage (pT2 and pT3), and associated to dynamic of alterations of immune cells (CD3+, CD4+, CD8+, CD19+, CD4+CD25+FoxP3+, CD3-CD16+CD56+, MDSC, CD8+CD69+), before surgery and three months following radical prostatectomy (RP). The concentrations of soluble PD-L1 and PD-1, were also compared between healthy individuals and patients with prostate cancer.

The inclusion criteria for healthy controls were as follows: (1) similarity in age to the patient group (range: 47–76 years). Exclusion criteria were as follows (1) history of malignancy diagnosis or treatment; (2) inflammatory conditions, immunosuppressive interventions, or autoimmune disease presence; (3) use of immunosuppressive drugs: prednisolone, cyclosporine, etc.

### 2.2 Blood sampling

Soluble levels of sPD-L1 and sPD-1 were assessed in the peripheral blood of all participants in this study. The evaluation was carried out for patients at two time points: 0–1 day before surgery and approximately three months (82–107 days) after radical prostatectomy. Healthy subjects underwent a single assessment. Additionally, specific immune cell populations were examined in patients prior to surgery and at the three-month postoperative mark. Blood samples were collected from all participants by venipuncture into BD Vacutainer^®^ tubes containing EDTA anticoagulant (BD Biosciences, San Jose, CA, USA). The tubes were gently shaken prior to the phenotypic staining procedure and subsequent flow cytometry analysis. Plasma for ELISA analysis was acquired through centrifugation, at 2500× g for 10 minutes and samples were stored at − 80°C until analysis of sPD-L1 and sPD-1 was performed. sPD-L1 and sPD-1 evaluation from cases and controls were processed simultaneously.

### 2.3 Analysis of soluble PD-L1 and PD-1

A commercially available human PD-L1 ELISA kit (Invitrogen, Thermo Fisher Scientific, Bender MedSystems GmbH Campus Vienna Biocenter, Vienna, Austria) was used to measure sPD-L1 protein concentrations in plasma. Similarly, a PD-1 human kit (Invitrogen, Thermo Fisher Scientific, Bender MedSystems GmbH Campus Vienna Biocenter, Vienna, Austria) was used to measure sPD-1 protein levels. The preparation of standards, samples, and all assay steps were conducted in accordance with the manufacturer’s instructions. Each sample was analyzed in duplicate, and the optical density was assessed at 450 nm using a BioTek Elx800 TM plate reader (BIO-Tek Instruments, Inc. PO Box 998, Highland Park Winooski, Vermont, USA).

### 2.4 Analysis of immune cells

Whole blood samples, were used for flow cytometry analysis and processed in accordance with the manufacturer’s guidelines. 100 μL of blood were added to each of the four flow cytometry tubes per patient and cell staining was conducted using the following antibodies. Tube 1: anti-CD25-PE/anti-CD4-FITC/anti-CD3-APC/anti-FoxP3-BV421™ (BioLegend, San Diego, CA, USA); tube 2: anti-CD8a-FITC/anti-CD69-APC/anti-CD3-BV510™ (BioLegend, San Diego, CA, USA); tube 3: anti-HLA-DR-PE/anti-CD14-FITC/anti-CD11b-BV421™/anti-CD33-APC (BioLegend, San Diego, CA, USA); tube 4: anti-CD56-PE/anti-CD16-APC/anti-CD3-FITC/anti-CD19-BV421™/anti-CD45-PerCP (BioLegend, San Diego, CA, USA). T regulatory cells were defined as CD4^+^ CD25^+^ FoxP3^+^, NK cells as CD3^-^ CD16^+^ CD56^+^, and total MDSCs as CD45^+^ CD3^−^ CD19^−^ CD56^−^ CD16^−^ HLA-DR^−^ CD33^+^ CD11b^+^. Cells were incubated with fluorescently labeled antibodies targeting cell surface markers, for 15 minutes in the darkness, to allow antibody binding. Subsequently, red blood cells were lysed for 15 minutes in the darkness using BD FACS Lysing solution (BD Biosciences, San Jose, CA, USA). Cells were washed twice to remove excess antibodies and lysing reagents in BD-Cell-Wash solution (BD Biosciences, San Jose, CA, USA) and then fixed in BD-Cell-Fix solution (BD Biosciences, San Jose, CA, USA). Immune components were analyzed by BD LSR II System flow cytometer (BD Biosciences, San Jose, CA, USA). Subset analysis was performed using BD FACSDivaTM Software (BD Biosciences, San Jose, CA, USA) with acquisition of a total of 20,000 events (29).

### 2.5 Statistics

The Shapiro-Wilks test was used to assess the normality of the data. Following that, Mann-Whitney U test with two-tailed hypothesis was employed to compare sPD-L1 and sPD-1 levels among patients and control group. Subsequently, Kruskal Wallis test was used to evaluate sPD-L1/sPD-1 levels between different patient’s groups. The relationship between continuous and categorical data variables was examined using a Spearman correlation test. Survival probabilities were estimated using the Kaplan-Meier method, and group comparisons were made using the log-rank test. The Cox proportional hazards model was used to estimate the hazard ratios for progression-free survival associated with sPD-1, sPD-L1, Gleason score, pathological stage, and PSA levels. A univariate Cox regression analysis was conducted, and variables with a p-value of less than 0.1 in the univariate survival analysis were incorporated into the multivariate analysis. The threshold between high and low sPD-L1 and sPD-1 concentrations was determined by logistic regression, followed by calculation of the Youden index to establish the cutoff values. All analyses were carried out in Python version 3.11.4 (Python Software Foundation), with statistical significance set at p <0.05.

## 3 Results

### 3.1 Characteristics of PCa population

A total of 88 patients with the pT2 and pT3 PCa diagnosis were included in our analysis. The median age of the patient group was 62.6 years, preoperative PSA levels ranged between 2.15 and 67.7 ng/ml and Gleason score was between 6 and 8 (grade group 1 to 4 respectively, according to ISUP). For 75 patients preoperative and postoperative sPD-L1 and sPD-1 pairs were determined to evaluate the dynamic of changes of soluble checkpoints after surgical treatment. Patient characteristics are summarized in **Table 1**.

**Table 1:** Characteristics of PCa patients.

Patient Characteristics	
No of patients (%)	88 (100%)
Age (years)	
<61	32 (36.4%)
61–65	26 (29.5%)
>65	30 (34.1%)
Preoperative PSA (ng/mL)	8.97 (2.15 – 67.7)
pT stage	
pT2	64 (72.2%)
pT3	24 (27.3%)
Gleason Grade	
Grade 1 [3 + 3]	24 (27.3%)
Grade 2 [3 + 4]	53 (60.2%)
Grade 3 [4 + 3]	10 (11.4%)
Grade 4 [4 + 4]	1 (1.1%)
Lymph node involvement	5 (5.7%)

### 3.2 Characteristics of control group

Eligible subjects (n = 41) were selected based on PSA level (<3 ng/ml), the subjects were at an average of 64.6 year (median 66), interquartile range (IQR) = 7. The control group was managed, and control samples were collected in collaboration with the biomedical research laboratory Rezus.lt. Characteristics of healthy subjects are summarized in **Table 2**.

**Table 2:** Characteristics of healthy subjects.

Control Characteristics	
**No of individuals (%)**	**41 (100%)**
Age (years)	
**<61**	**9 (22%)**
**61–65**	**11 (26.8%)**
**>65**	**21 (51.2%)**
**PSA (ng/mL)**	**1.19 (0 – 2.96)**

### 3.3 sPD-L1 and sPD-1 in PCa patients and healthy subjects

**Figure 1:**
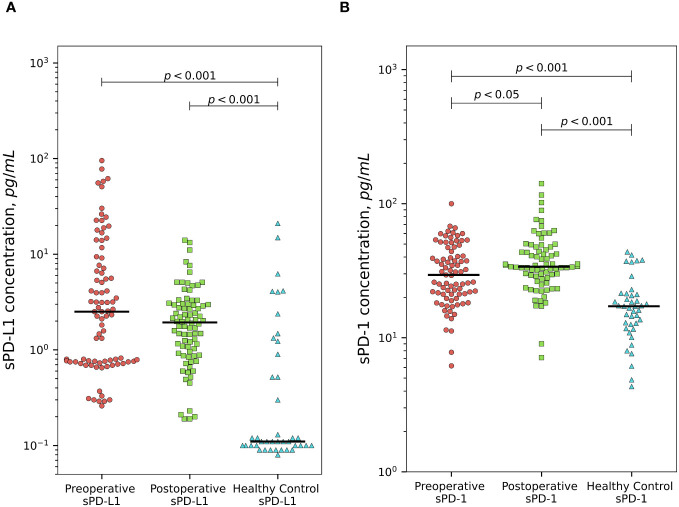
Comparison of sPD-L1 and sPD-1 levels between prostate cancer patients and healthy subjects. PCa patients demonstrates significantly higher preoperative and postoperative plasma sPD-L1 levels comparing to healthy individuals (p < 0.001). sPD-L1 results in statistically unsignificant postoperative decline. **(A)** Presurgical and postsurgical plasma sPD-1 levels in PCa patients are significantly higher than in healthy controls (p < 0.001). sPD-1 demonstrates statistically significant postoperative increase (p < 0.05) **(B)**.

Plasma sPD-L1 levels were significantly higher comparing to healthy control group (median 0.11 pg/ml) both in preoperative (median 2.51 pg/ml) and postoperative (median 1.94 pg/ml) groups of PCa patients (p < 0.001). sPD-1 concentrations at baseline (median 29.44 pg/ml) and after surgical treatment (median 33.89 pg/ml) were significantly higher comparing to healthy control group (median 17.18) p < (0.001). sPD-L1 exhibits a statistically insignificant postoperative decline, whereas sPD-1 demonstrates a statistically significant postoperative increase (p < 0.05). sPD-L1 and sPD-1 values in the patients and in the controls are shown in **Figure 1**.

**Figure 2:**
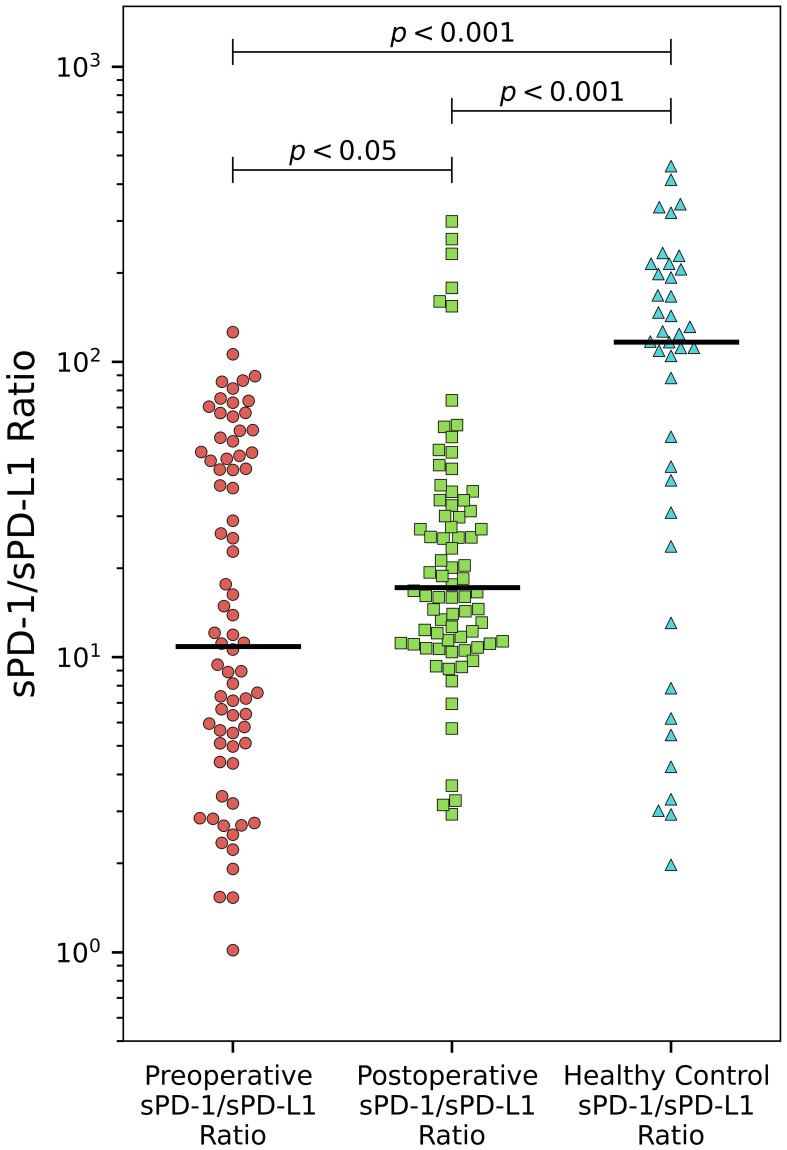
Alteration of sPD-/sPD-L1 ratio in PCa. The sPD-1/sPD-L1 ratio demonstrates significant increase after surgery (p < 0.05). Both preoperative and postoperative patient groups differ significantly compared to healthy controls (p < 0.001).

The sPD-1/sPD-L1 ratio exhibited statistically significant differences between both preoperative and postoperative patient groups compared to the control group. Postoperatively, we observed a notable rise in the sPD-1/sPD-L1 ratio, increasing from a median of 10.9 to 17.2 (p < 0.05). Remarkably, the control group had the highest median among all three groups at 116.7 (**Figure 2**).

### 3.4 The association of sPD-L1 and sPD-1 with clinicopathological findings of PCa

**Figure 3:**
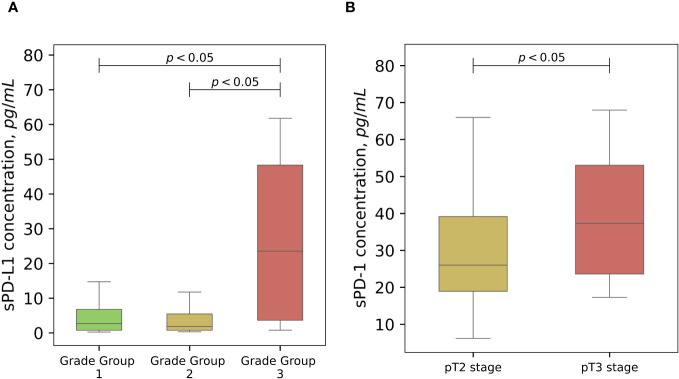
The associations of soluble PD-L1 and PD-1 with cancer progression. Variations of sPD-L1 concentration in the peripheral blood of PCa patients differs significantly (p < 0,05) according to Gleason grade groups, grade 3 group shows significant association with highly elevated levels of sPD-L1 **(A)**. Pretreatment sPD-1 levels demonstrate higher values in clinicopathological staging of PCa (p < 0.05) **(B)**.

Statistically significant association between baseline sPD-L1 and Gleason score was observed. Patients with grade group 3 has a significantly higher levels (median 23.5 pg/ml) of circulating PD-L1 comparing to grade group 1 (median 2.5 pg/ml) and grade group 2 (median 2.2 pg/ml) (p < 0.05), (**Figure 3A**). The grade group 4 was not analyzed because the number of subjects in that group was too small to obtain a reliable result. A statistically significant (p < 0.05) association between baseline sPD-1 concentration and advanced PCa stage was obtained (**Figure 3B**).

### 3.5 Prognostic and predictive value of sPD-L1 and sPD-1 in PCa

**Figure 4:**
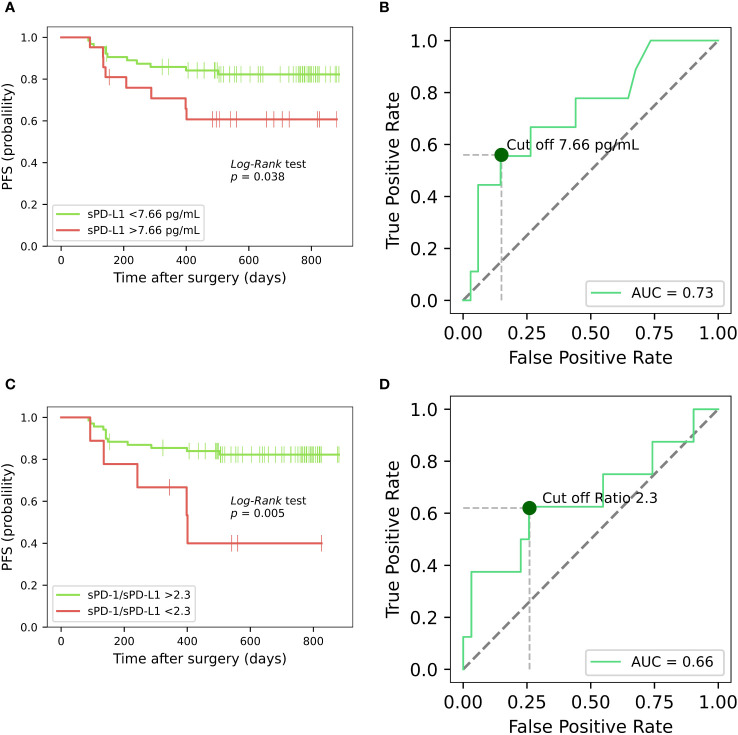
Kaplan-Meier analysis on the progression-free survival of prostate cancer patients, based on sPD-L1 and sPD-1/sPD-L1 ratio. Kaplan-Meier curves illustrates the progression-free survival of PCa patients based on high and low presurgical concentrations of sPD-L1, revealing that low pretreatment sPD-L1 levels are associated with prolonged PFS (p < 0.05) **(A)**. ROC curve for preoperative plasma concentrations of sPD-L1 for PFS prediction in PCa **(B)**. Kaplan-Meier analysis based on sPD-1/sPD-L1 ratio reveals that patients exhibiting a lower sPD-1/sPD-L1 ratio were correlated with an elevated probability of disease progression (p < 0.05) **(C)**. ROC curve of sPD-1/sPD-L1 ratio as classifier PFS in PCa **(D)**.

For progression free survival analysis, threshold of 7.66 pg/ml (specificity 85%, sensitivity 56%, AUC = 0.73) was established for preoperative plasma concentrations of sPD-L1, using receiver operating characteristic (ROC) curves (**Figure 4B**). PFS was compared between the low (< 7.66 pg/ml) and high (≥ 7.66 pg/ml) sPD-L1 groups by Kaplan-Meier analysis and log rank tests. Kaplan–Meier analysis revealed that postoperative PFS tended to be shorter in the high sPD-L1 group (n = 21) than in the low sPD-L1 group (n = 64) (p < 0.05) (**Figure 4A**). The difference in PFS survival between the groups was notable at 2 years post-surgery, with rates of 82% in the < 7.66 pg/mL group and 61% in the ≥ 7.66 pg/ml group. ROC analysis was conducted to assess the efficacy of sPD-1 as a classifier for PFS. However, sPD-1 was determined to be an ineffective classifier for PFS (AUC = 0.47). Subsequently, evaluation of PFS between the low (< 18.22 pg/ml) and high (≥ 18.22 pg/ml) sPD-1 groups was performed using Kaplan-Meier analysis and log-rank tests. Kaplan–Meier analysis showed that the low sPD-1 group (n = 15) as well as high sPD-1 group (n = 66) had no statistical significance for PFS estimation in PCa patients (Data not shown).

The potential of prognostic biomarkers for predicting PFS in PCa patients was investigated using Cox proportional hazard analysis. While the high sPD-L1 concentrations group exhibited significance in the univariate analysis, it failed to maintain significance in the multivariate model, suggesting it may not act as an independent predictor. Conversely, sPD-1 concentrations (both continuous and categorized) did not show significant associations with survival outcomes in univariate analysis, suggesting their reliability as predictors of PFS in this context may be limited. The results of the Cox proportional hazard analysis are summarized in **Tables 3**, **4**.

**Table 3:** The results of Cox proportional hazard, univariate analysis.

	Progression-free survival	
	Hazard Ratio (95% CI)	*P-value*
Gleason Grade Group		
Grade Group 1	Ref.	
Grade Group 2	6.95 (0.9–53)	0.061
Grade Group 3	31.43 (3.9–252)	0.001
**PSA, ng/mL**	1.04 (1.01–1.07)	0.005
pT stage		
pT2	Ref.	
pT3	11.2 (4.4–28.5)	*p*<0.001
sPD-1		
<18.22 pg/ml	Ref.	
>18.22 pg/mL	1.23 (0.4–4.3)	0.74
sPD-L1		
<7.66 pg/mL	Ref.	
>7.66 pg/mL	2.5 (1.02–6.3)	0.045
**sPD-1, pg/mL**	1.001 (0.98–1.03)	0.93
**sPD-L1, pg/mL**	1.015 (1–1.03)	0.08
sPD-1/sPD-L1 ratio		
<2.3	Ref	
>2.3	0.25 (0.09–0.72)	0.01
**sPD-1/sPD-L1, ratio**	1.0 (0.98–1.01)	0.58

**Table 4:** The results of Cox proportional hazard, multivariate analysis.

Variable	Model 1 sPD-L1 pg/mL (continuous)		Model 2 sPD-L1 (categorical)		Model 3 sPD-1/sPD-L1 ratio (categorical)	
	Hazard Ratio (95% CI)	*P-value*	Hazard Ratio (95% CI)	*P-value*	Hazard Ratio (95% CI)	*P-value*
Gleason Grade Group						
Grade Group 1	Ref.		Ref.		Ref.	
Grade Group 2	15.6 (0.9–266)	0.06	15.9 (0.9–273)	0.06	73.3 (0.5–9800)	0.08
Grade Group 3	71.3 (3.7–1376)	0.005	56.9 (3.2–1018)	0.006	131.75 (1–16790)	0.049
**PSA, ng/mL**	1.05 (1.004–1.11)	0.03	1.06 (1.05–1.11)	0.03	1.09 (1.0–1.19)	0.053
pT stage						
pT2	Ref.		Ref.		Ref.	
pT3	5.7 (1.9–17.4)	0.002	5.3 (1.7–16.7)	0.004	5.7 (1.9–17.8)	0.002
**sPD-L1, pg/mL**	1.0 (0.96–1.02)	0.5	–	–	–	
sPD-L1						
<7.66 pg/mL	–		Ref.		–	
>7.66 pg/mL	–		0.96 (0.3–3.2)	0.95	–	
sPD-1/sPD-L1 ratio						
<2.3	–		–		Ref	
>2.3	–		–		0.51 (0.11–2.3)	0.39

### 3.6 The sPD-1/sPD-L1 ratio for PFS prediction

Based on the ROC curve analysis, a preoperative sPD-1/sPD-L1 ratio of 2.3 was identified (specificity 90%, sensitivity 56%, AUC = 0.66) (**Figure 4D**). Patients with a lower sPD-1/sPD-L1 ratio were associated with a higher probability of disease progression (p < 0.05). One year PFS was 75% in the group with < 2.3 sPD-1/sPD-L1 ratio, contrasting with 84% in the > 2.3 ratio group. Probabilities of PFS differed even more between groups at 2 years after surgery. At 2 years postoperatively, the < 2.3 sPD-1/sPD-L1 ratio group had a 45% probability of PFS, while the > 2.3 ratio group had an 81% probability (**Figure 4C**). In Cox analysis, categorizing the preoperative sPD-1/sPD-L1 ratio into high and low ratio groups yielded comparable results to single sPD-L1 (**Tables 3**, **4**). However, in univariate analysis, similar to continuous sPD-L1, the continuous sPD-1/sPD-L1 ratio was not found to be significant. Furthermore, the postoperative ratio of sPD-1/sPD-L1 showed no prognostic significance in Cox regression and performed poorly as a classifier for PFS in ROC analysis.

### 3.7 The changes of sPD-L1 and sPD-1 after radical prostatectomy in PCa

**Figure 5:**
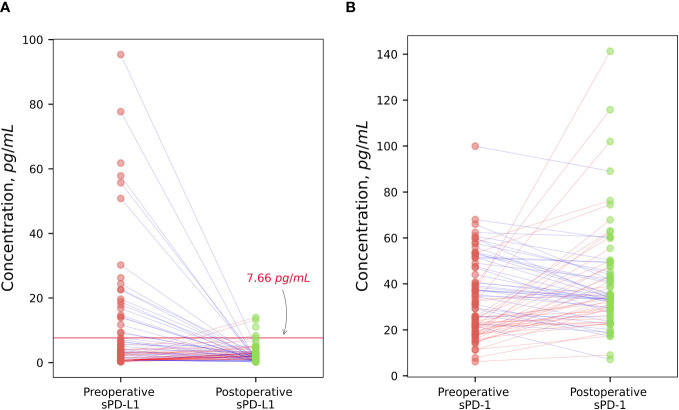
The dynamic of changes of sPD-L1 and sPD-1 after surgical treatment. Radical prostatectomy in individualized scatterplot analysis demonstrated statistically significant decrease (p < 0.001) of high baseline sPD-L1 level (> 7.66 pg/ml), and statistically significant increase (p < 0.05) of low preoperative sPD-L1 levels (< 7,66 pg/ml), whereas the postoperative variation of sPD-L1 in the whole group of patients varied and was insignificant **(A)**. Individualized sPD-1 response to surgical tumor removal showed statistically significant increase (p < 0.05) **(B)**.

There was no significant difference between preoperative values of sPD-L1 and after radical prostatectomy in the whole patient’s group of PCa (p = 0.12). Scatterplot analysis revealed that the overall mean of sPD-L1 in the patient group decreased after surgery. According to detailed analysis, - individuals whose estimated initial sPD-L1 level was high (> 7.66 pg/ml) showed a statistically significant postoperative decrease (p < 0.001) and whose presurgical sPD-L1 level was low (< 7.66 pg/ml) showed a statistically significant postoperative increase (p < 0.05) (**Figure 5A**).

Tumor excision resulted in a noticeable sPD-1 increase (p < 0.05). According to scatterplot analysis post-operative concentrations of sPD-1 in PCa patients changed in variable way, however, the overall group mean concentrations after radical tumor removal were increased (**Figure 5B**).

### 3.8 The interplay between sPD-L1 and sPD-1 and circulating immune cells in PCa

There was no significant interplay between soluble PD-L1 and PD-1 and immune subsets CD3+, CD4+, CD8+, CD19+, CD3-CD16+CD56+, CD8+CD69+.

**Figure 6:**
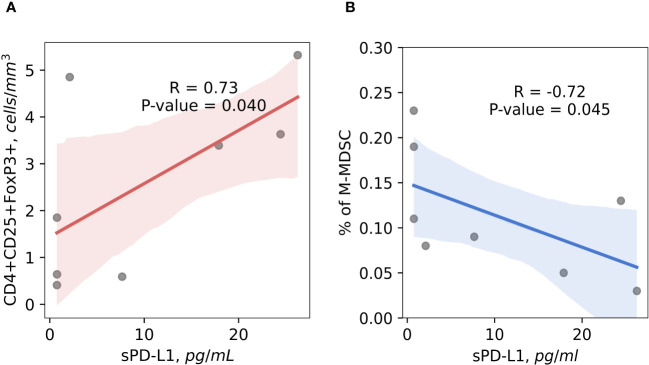
The correlation between preoperative circulating immunosuppressive cells and sPD-L1. Preoperative CD4+CD25+FoxP3+ Tregs cells positively correlates with sPD-L1 in patients with occurred BCR, suggesting the contribution for cancer progression (R = 0.73) (p < 0.05) **(A)**. Baseline percentage of M-MDSC cells and sPD-L1 shows significant inverse correlation in the peripheral blood of prostate cancer patients with BCR occurred, indicating that monocytic MDSC are not associated with sPD-L1 production in complicated disease (r = -0.72) (p < 0.05) **(B)**.

A high positive correlation (r = 0.73) (p < 0.05) between immunosuppressive CD4+CD25+FoxP3+ regulatory T cells and presurgical sPD-L1 was established in the patients with occurred BCR (**Figure 6A**). Among patients who have experienced BCR, was found a strong inverse correlation (r = -0.72) (p < 0.05) between preoperative sPD-L1 and percentage of M-MDSC (**Figure 6B**). In patients with favorable postoperative course of disease no correlations were found between sPD-L1 and Tregs (r = -0.02) (p = 0.86) and percentage of M-MDSC (r = -0.21) (p < 0.10) (Data not shown).

## 4 Discussion

### 4.1 Soluble PD-L1 and PD-1 in prostate cancer and healthy subjects

In present study, we performed a comprehensive analysis of circulating sPD-L1 and sPD-1 levels in a cohort comprising both PCa patients and healthy individuals. Prior to our investigation, - circulating sPD-L1 and sPD-1 levels have never been studied in PCa of the European male population. Levels of sPD-L1 and sPD-1 and their prognostic value in PCa were analyzed only in two trials – in African and in USA men populations (30, 31). Both studies showed elevated concentrations of these molecules in patients with metastatic castration-resistant prostate cancer (mCRPC) compared to healthy controls. Notably, these studies focused on a more aggressive form of prostate cancer compared to our research. Additionally, ADT was administered during the examination in the African male population and no surgical treatment was utilized in either study. Furthermore, it is recognized that prostate cancer affects African men nearly twice as frequently as European men (30), due to various genetic mutations (32).

Our investigation revealed elevated levels of soluble PD-L1 and PD-1 in the plasma of PCa patients, both before and after surgical treatment, compared to healthy controls (**Figures 1A, B**). sPD-L1 is found in healthy humans and significantly increases in the blood of aged healthy individuals (33), the 51–70 years old people have the highest level of sPD-L1 (13). Our control group, meticulously selected, based on medical history and drug usage, closely mirrors the study population’s age profile and the peak of sPD-L1 concentration in healthy individuals. This makes it an ideal reference for comparing sPD-L1 levels between PCa patients and healthy men. Elevated concentrations of circulating sPD-L1 were found in many cancers compared to controls, - in two separate studies of gastric cancer (GC) (34, 35) in hepatocellular carcinoma (HCC) (36) lung adenocarcinoma (37) clear cell renal cell carcinoma (ccRCC) (12), in different types of carcinomas (38), ovarian cancer (OC) (39), glioma (40, 41).

sPD-1 levels tend to be higher in various cancers compared to those in healthy subjects as well. Elevated levels were found in different subtypes of lymphoma (42), lung adenocarcinoma (37) and glioma (41).

We observed no significant correlation between PSA levels and sPD-L1 or sPD-1 in our patient’s group. Furthermore, when stratifying patients based on median sPD-L1 and sPD-1 levels or utilizing logistic regression to define sPD-L1 groups, no significant differences in PSA levels were detected. These findings suggest that sPD-1 and sPD-L1 may hold promise as complementary biomarkers for prostate cancer screening, potentially enhancing the accuracy of screening alongside PSA testing.

### 4.2 The implications of sPD-L1 in prostate cancer

To assess the tumor-dependent relationship of sPD-L1 and sPD-1, we compared the preoperative and postoperative dynamics in prostate cancer patients. Our results showed no statistically significant decrease in sPD-L1 levels following tumor excision across the entire patient’s cohort. Therefore, the individualized response to radical tumor removal demonstrates different directions of sPD-L1 changes in high and low groups of sPD-L1. Remarkably, patients with initially high sPD-L1 levels exhibited a statistically significant decrease postoperatively, whereas those with low preoperative sPD-L1 levels demonstrated a statistically significant postsurgical increase (**Figure 5A**).

Surgical tumor removal has been associated with decreased levels of sPD-L1 in various cancers. In glioma patients, postoperative sPD-L1 levels were significantly lower than preoperative levels (41). Non-small cell lung cancer (NSCLC) patients undergoing radical surgery exhibited a significant increase in sPD-L1 one month post-surgery, followed by a slight decrease at three months (14), suggesting a poor link to tumor removal. While we lack serial data for postoperative changes in PCa patients, comparing with NSCLC studies, suggests that three months may be sufficient to assess the tumor and sPD-L1 dependence. These findings lead us to hypothesize that elevated baseline sPD-L1 levels may relate to tumor secretion. Additionally, sources other than tumor cells, might contribute to the relatively modest sPD-L1 levels observed in peripheral blood in PCa. Furthermore, Kaplan–Meier analysis based on high and low baseline sPD-L1 levels, showed contrasting predictions for PFS. This supports the notion that sPD-L1 in prostate cancer originates not only from tumor cells but also from other cellular sources (**Figure 4A**).

Low PD-L1 expression in the TME of prostate cancer can complicate the association with sPD-L1. However, PD-L1 expression by tumor cells in prostate cancer correlates with tumor stage, Gleason score, lymph node or distant metastases, surgical margin positivity and other. PD-L1 positivity rates vary in primary prostate cancers and different metastatic sites within the same patient (43), highlighting high heterogeneity of PCa tumor (44). Despite high membranous PD-L1 expression, some studies found no significant correlation between sPD-L1 and tissue PD-L1 (34).

To the best of our knowledge the values of soluble PD-L1 and PD-1 were not further investigated in association with PFS in PCa. Given the cold immune subtype of prostate tumor, Kaplan Meier analysis in our study revealed shortened PFS correlation with high baseline sPD-L1 values in PCa, as in many other tumors characterized by non-immunologically cold TME such as HCC (45), gastric cancer (46, 47), breast cancer (BC) (48) glioma (41) and several NSCLC studies (14, 49, 50).

The Cox proportional hazard test for PFS, initially identified sPD-L1 as a significant factor in the univariate analysis. However, upon conducting multivariate Cox analysis, sPD-L1 lost its significance. This suggests that sPD-L1 does not contribute additional prognostic value beyond established clinical factors such as Gleason score, PSA level, and disease stage in PCa. These results imply that while sPD-L1 may have shown promise in isolation, it does not offer incremental prognostic insight beyond conventional clinical parameters routinely used in PCa prognosis. The significance found in the univariate Cox model, but not in the multivariate Cox model, may also be attributed to the relatively small sample size of the group with disease progression (n = 23). Baseline sPD-L1 was good marker of tumor recurrence in BC (48) and of PFS in metastatic ccRCC patients, treated with sunitinib (51). A previous mentioned studies on soluble immune checkpoints in African and American populations did not explore their association with PFS.

The correlation between circulating sPD-L1 and overall survival (OS) has been observed across various cancers. Patients with high preoperative serum sPD-L1 levels showed significantly lower OS compared to those with low levels in gastric cancer (34, 46) and metastatic pancreatic cancer (52). Huang’s meta-analysis (53) and another multicancer study by Scirocci (9), both found that elevated sPD-L1 levels were associated with worse survival outcomes. Pretreatment sPD-L1 levels were prognostic indicators for OS in patients with biliary tract cancer undergoing palliative chemotherapy (54). No deaths occurred during the follow-up period in our investigation.

Based on our investigation, disease progression correlates with elevated levels of circulating sPD-L1. Significantly elevated sPD-L1 levels were associated with higher Gleason scores, particularly with 4 + 3. This association was also observed among African men, as reported by Katangole (30), although their study showed a higher percentage (43.86%) of advanced PCa cases with Gleason scores of 8–10, whereas in our study, only one person had a Gleason score of 8. The predominant Gleason scores in our group were 6–7 (**Table 1**). The correlation between higher grade (group 3) and sPD-L1 suggests that sPD-L1 may contribute to the aggressiveness of the disease in our study’s PCa patients. Consistent with our findings, the association of sPD-L1 with disease advancement parameters has been identified in various other tumors: in aggressive bladder cancer (55), advanced (ccRCC) (12), and gastric cancer (35). Baseline sPD-L1 has proven to be a reliable tumor marker in metastatic breast cancer (48) and has been linked to rapid metastatic progression in metastatic ccRCC (51), as well as to the size of metastases in colorectal cancer (56). Elevated initial sPD-L1 levels have been associated with poorer prognosis in ccRCC (57), soft tissue sarcomas (28), pancreatic adenocarcinoma (58), lung cancer (49, 59), hepatocellular carcinoma (36), and lower-grade glioma (40). Moreover, high pretreatment sPD-L1 levels have been linked to low disease control rates in various advanced solid tumors, including melanoma, NSCLC and other (60). As in our study, higher sPD-L1 score values are linked with increased tumor invasiveness, potentially aiding in the identification of high-risk patients who could benefit from prostate biopsy. This approach could help reduce the number of unnecessary biopsies in PCa.

### 4.3 The impact of sPD-1 in prostate cancer progression

We observed a significant increase in sPD1 levels following tumor excision. Elevated sPD-1 levels following cancer treatment, including surgery, are believed to be associated with favorable outcomes (5). Postreatment sPD-1 levels varies across different cancers: notably increased post immunotherapy vaccine application in mCRPC cases (31) and after anti-PD-1 antibody therapy in solid tumors (61). However, glioma patients had lower postoperative sPD-1 levels compared to preoperative levels (41). Higher post-ICI monotherapy sPD-1 levels were linked to longer overall survival in NSCLC patients (49) and advanced EGFR-mutated NSCLC patients treated with erlotinib (62). Despite the significant elevation of sPD-1 levels following radical prostatectomy, suggesting potential better outcomes, our investigation found no correlation between prognosis and higher postoperative circulating sPD-1 levels. Hypothetically, the postoperative increase in soluble PD-1 levels could be attributed to various factors and mechanisms. Studies suggest sPD-1’s potential to counteract the immunosuppressive effects of PD-1/PD-L1, restoring T-cell function and enhancing antitumor immunity (11, 63). On the other hand, sPD-L1 has been shown to suppress peripheral T lymphocytes (5, 12). The decrease in sPD-L1 post-surgery aligns with the natural T lymphocyte recovery seen with radical tumor resection (64). Overall, tumor excision removes the immune system’s suppressive burden, potentially leading to immune restoration and an increase in sPD-1 levels.

We discovered a statistically significant association between baseline sPD-1 concentration and advanced cancer stage, suggesting a potential correlation between sPD-1 levels and poorer prognosis in PCa. The higher concentration of sPD-1 in pT3 lesions could be useful for doctors to more accurately determine the stage of the cancer. In metastatic ccRCC higher concentrations of sPD-1 tended to correlate with advanced cancer stage as well (51). For patients with metastatic colorectal cancer (mCRC), soluble PD-1, similar to soluble PD-L1, was linked to clinically worse levels of various peripheral blood parameters and metastatic tumor burden (56) and predicted systemic inflammation in pancreatic cancer (65). Although our data reflects finding between pretreatment sPD-1 levels and tumor progression in other cancers, the precise mechanism of the role of sPD-1 remains unclear. We hypothesize that as cancer progresses, there might be an increase in soluble PD-1 as part of the complex interplay between the tumor and the immune system. The tumor microenvironment can release factors that promote the shedding of PD-1 from cell surfaces, resulting in elevated soluble PD-1 levels, particularly since the exact source of sPD-1 is still unknown. Additionally, tumor-associated inflammation can trigger an immune response, leading to higher soluble PD-1 levels as part of immune regulation. Some studies have confirmed the association of sPD-1 with systemic inflammation in the context of cancer progression (65). Further studies are needed to determine the exact mechanism that underlies the connection between malignancy and sPD-1 (11).

Our findings regarding initial sPD-1 levels and PFS did not show statistical significance and according to results of Cox analysis, sPD-1 may not be a reliable predictor of PFS. Contrary, in other cancer studies it has been demonstrated that untreated cancer patients with elevated sPD-1 will have unfavorable survival outcomes (5, 11). High sPD-1 concentrations predict reduced PFS duration in glioma (41) NSCLC (50) mCRC (56) and pancreatic adenocarcinoma (58). Poor survival in diffuse large B cell lymphoma was indicated by a correlation between high initial sPD-1 levels and the PD1+ T cells infiltrating the tumor (66). In contrast a high level of sPD-1 correlated with prolonged PFS in HCC (45). In metastatic ccRCC patients receiving sunitinib (51) treatment and patients treated with nivolumab and ipilimumab in melanoma, sPD-1 levels were significant predictive markers of PFS (67).

### 4.4 The sPD-1/sPD-L1 ratio for PCa prognosis prediction

PD-1 and sPD-L1 may exert opposing functions potentially creating either an active (11, 63) or immunosuppressive (5, 12, 38, 68) environment depending on their respective concentrations. In our study, the sPD-1/sPD-L1 ratio was significantly different before surgery and after surgery compared with the control group, which exhibited highest values (**Figure 2**). We found a statistically significant association between initial sPD-1/sPD-L1 ratio and PFS. Patients exhibiting a lower sPD-1/sPD-L1 ratio were associated with a shorter PFS (p < 0.05). Similar results were observed for patient survival by sPD-1/PD-L1 ratio in melanoma treated with immune checkpoint blockade (69), as well as in the context of low sPD-1 and high sPD-L1 combination for PD-1 antibody monotherapy across various cancers (70). In our study, we observed a significant increase in the sPD-1/sPD-L1 ratio after surgery in the entire prostate cancer (PCa) population. This supports preoperative findings indicating a correlation between a higher baseline sPD-1/sPD-L1 ratio and improved prognosis. The postoperative rise in the sPD-1/sPD-L1 ratio suggests a favorable prognosis, particularly considering the highest ratio observed in healthy subjects. In the melanoma study, patients undergoing immunotherapy exhibited around a 30% decrease in mortality risk at a specific time point among those with elevated ratios of sPD-1/sPD-L1 (69). However, in our study, the sPD-1/sPD-L1 ratio demonstrated significance in Cox univariate analysis for PFS but lost its significance in the multivariate analysis, suggesting that it does not offer additional prognostic value beyond established clinical factors in PCa prognosis. Moreover, while the preoperative ratio could distinguish patients more likely to experience disease progression, we did not observe any discernible pattern in the change (increase or decrease) of the sPD-1/sPD-L1 ratio and prognosis.

### 4.5 The relationship between sPD-L1 and immunosuppressive cells

There are multiple ways in which cancer cells can suppress the immune system’s ability to fight tumors. These include increasing the levels of immune checkpoint proteins and enhancing the immunosuppressive effects of regulatory T cells and MDSCs infiltrating the TME (71). To analyze how the interactions of sPD-L1 and sPD-1 with circulating immune cells affect tumor progression, we further explored the relationships between sPD-L1, sPD-1, and circulating immune cells in both favorable and unfavorable disease outcomes. Preoperative levels of immunosuppressive cells such as Tregs and MDSC, showed notable correlations with baseline sPD-L1 levels in postoperative BCR patients. Conversely, no correlations were observed between sPD-L1 and immune cell populations in patients with a favorable disease course. No notable correlations were found between sPD-L1 and sPD-1 levels and immune cell populations like CD3+, CD4+, CD8+, and NK cells, which typically exhibits antitumor effects in whole PCa patients population.

Soluble PD-L1 and PD-1 were found to impact clinically worse laboratory parameters in mCRC, as indicated by Dank (56). Additionally, Castello (72) discovered an association between metabolic tumor burden and sPD-L1 levels in NSCLC, while Kruger (65) revealed a link between sPD-L1 and sPD-1 with systemic inflammation in pancreatic cancer. These findings suggest a systemic effect of these soluble molecules on unfavorable prognosis and cancer progression. However, further studies investigating the effects of soluble PD-L1 and PD-1 on disease progression beyond intrinsic clinical findings are still limited.

Based on the possible systemic effect of soluble PD-L1 and PD-1, our analysis revealed a positive correlation between sPD-L1 and immunosuppressive circulating CD4+CD25+FOXP3+ T regulatory cells in PCa patients with an unfavorable course of disease, suggesting potential role of this interaction in disease progression and upregulated immunosuppressive activity. Several cancer studies support PD-L1’s involvement in T regulatory cell proliferation and immunosuppression. PD-L1 regulates induced Treg cells development and functionality (73), later these cells have been shown to be induced and sustained by PD-L1 in glioblastoma (74). Acute myeloid leukemia (AML) expressing PD-L1, may enhances Treg cells expansion, which, in turn, stimulates AML cell growth via production of specific interleukins (75). Additionally, sPD-L1 was found to induce B regulatory cell differentiation and regulate Treg induction through CD19+ B cells (76). Recent data from Liang (38) suggests circulating sPD-L1 beyond the TME promotes cancer growth. Our findings, along with these studies, suggest sPD-L1 enhances the immunosuppressive effects of T regulatory cells, potentially driving the progression of cancer.

MDSCs, known to contribute to sPD-L1 production, in addition to tumor cells (77). However, it remains unclear whether monocytic (M-MDSC) or granulocytic (G-MDSC) subtypes predominantly produce sPD-L1 (60). Studies in tumor-bearing mice have shown higher percentages of PD-L1+ G-MDSCs and M-MDSCs compared to tumor-free mice, with M-MDSCs exhibiting the highest proportion of PD-L1 expression (78). Oh’s study suggests that sPD-L1 originates primarily from G-MDSCs (60). In a mouse colon cancer model, the tumor microenvironment had the highest concentration of PD-L1+ MDSCs compared to peripheral blood and secondary lymphoid organs (78). Conversely, ovarian cancer showed a strong link between PD-L1+ M-MDSCs and sPD-L1 in the bloodstream, suggesting sPD-L1 as a potential marker for monitoring PD-L1+ myeloid cells in untreated OC without invasive procedures (39). Our study found an inverse correlation between baseline sPD-L1 levels and M-MDSC percentage in patients with BCR, suggesting M-MDSCs are not a major source of sPD-L1 in PCa with unfavorable course. Additionally, patients with high baseline sPD-L1 levels were associated with shorter PFS, (including BCR) and showed significant postoperative decreases, indicating a link between high sPD-L1 and tumor. This supports our hypothesis that high sPD-L1 levels may be linked to tumor secretion. We aimed to link the immune cells we studied with sPD-L1, anticipating that they might serve as an additional source of sPD-L1; however, we did not find any such associations.

## 5 Conclusion

New cancer biomarkers may be provided by the implementation of the easily, into clinical practice, introducible sPD-L1 and sPD-1, as these have demonstrated significance in tumor prognosis and possible systemic effect for cancer proliferation. In our study, sPD-L1 and sPD-1 levels were higher in PCa patients compared to healthy individuals. High initial sPD-L1 concentrations correlated with poorer PFS, as well as a low sPD-1/sPD-L1 ratio and has emerged as a valuable prognostic marker for PCa. Additionally, a strong link between sPD-L1 and CD4+CD25+Foxp3+ regulatory T cells in BCR cases was observed, indicating sPD-L1’s systemic impact on tumor progression. We found an inverse relationship between M-MDSC percentage and sPD-L1 in BCR patients. This, along with high sPD-L1 levels associating with shortened PFS and postoperative decrease, suggests M-MDSC might not be the source of sPD-L1 in PCa patients. Our findings suggest sPD-L1 elevation relates to tumor progression and initial concentration of sPD-L1 is appropriate to predict the course of disease. Although sPD-L1 may be linked with other PCa diagnostic tools utilized in clinical settings, its convenient accessibility facilitates the implementation of more thorough screening protocols. Future directions, - more detailed studies of the interactions of PD-L1 and sPD-1 with immune cells in other tumors and the association of exposure with adverse prognosis in larger cohorts are needed, as well as detailed studies of the associations with other parameters supporting their systemic effect for tumor progression and origin. In conclusion, - the results of this study suggest that pretreatment plasma sPD-L1 concentrations can be used for prognosis prediction and could be helpful in biopsy decisions in prostate cancer.

## 6 Limitations of the study

There were some limitations in the current study. First, this is a single-center study, with relatively small study cohort. Second, the profile of immune cells has not been studied widely enough.

## Data availability statement

The raw data supporting the conclusions of this article will be made available by the authors, without undue reservation.

## Ethics statement

The studies involving humans were approved by Vilnius Regional Ethics Committee on Biomedical Research, (Vilnius, Lithuania). The studies were conducted in accordance with the local legislation and institutional requirements. The participants provided their written informed consent to participate in this study.

## Author contributions

MZ: Data curation, Investigation, Resources, Validation, Visualization, Writing – original draft. ZS: Data curation, Formal analysis, Investigation, Validation, Visualization, Writing – review & editing. PB: Data curation, Investigation, Validation, Writing – review & editing. ND: Data curation, Investigation, Writing – review & editing. AM: Data curation, Investigation, Writing – review & editing. GZ: Conceptualization, Writing – review & editing. JJ: Project administration, Writing – review & editing. DC: Funding acquisition, Resources, Supervision, Validation, Visualization, Writing – review & editing. VP: Project administration, Supervision, Validation, Visualization, Writing – review & editing, Conceptualization.
